# Facile Synthesis of Low-Cost Copper-Silver and Cobalt-Silver Alloy Nanoparticles on Reduced Graphene Oxide as Efficient Electrocatalysts for Oxygen Reduction Reaction in Alkaline Media

**DOI:** 10.3390/nano12152657

**Published:** 2022-08-02

**Authors:** Jadranka Milikić, Sara Knežević, Stevan Stojadinović, Mabkhoot Alsaiari, Farid A. Harraz, Diogo M. F. Santos, Biljana Šljukić

**Affiliations:** 1 University of Belgrade, Faculty of Physical Chemistry, Studentski trg 12-16, 11158 Belgrade, Serbia; biljka@ffh.bg.ac.rs; 2 Faculty of Chemistry, University of Belgrade, Studentski trg 12-16, 11000 Belgrade, Serbia; packdatc@gmail.com; 3 University of Belgrade, Faculty of Physics, Studentski trg 12-16, 11000 Belgrade, Serbia; sstevan@ff.bg.ac.rs; 4 Promising Centre for Sensors and Electronic Devices (PCSED), Advanced Materials and Nano-Research Centre, Najran University, Najran 11001, Saudi Arabia; mamalsaiari@nu.edu.sa (M.A.); faharraz@nu.edu.sa (F.A.H.); 5 Department of Chemistry, Faculty of Science and Arts at Sharurah, Najran University, Najran 11001, Saudi Arabia; 6 Nanomaterials and Nanotechnology Department, Central Metallurgical Research and Development Institute (CMRDI), Helwan 11421, Cairo, Egypt; 7 Center of Physics and Engineering of Advanced Materials, Laboratory for Physics of Materials and Emerging Technologies, Chemical Engineering Department, Instituto Superior Técnico, Universidade de Lisboa, 1049-001 Lisbon, Portugal

**Keywords:** oxygen reduction reaction, hydrogen peroxide reduction reaction, nanoparticles, CuAg alloy, CoAg alloy, reduced graphene oxide

## Abstract

Copper-silver and cobalt-silver alloy nanoparticles deposited on reduced graphene oxide (CuAg/rGO and CoAg/rGO) were synthesized and examined as electrocatalysts for oxygen reduction reaction (ORR) and hydrogen peroxide reduction reaction (HPRR) in alkaline media. Characterization of the prepared samples was done by transmission electron microscopy (TEM), Fourier-transform infrared spectroscopy (FTIR), Raman spectroscopy, X-ray diffraction analysis (XRD), and scanning electron microscopy with integrated energy-dispersive X-ray spectroscopy (SEM-EDS). CuAg/rGO and CoAg/rGO nanoparticles diameter ranged from 0.4 to 9.2 nm. The Ag loading was ca. 40 wt.% for both electrocatalysts, with that for Cu and Co being 35 and 17 wt.%, respectively. CoAg/rGO electrocatalyst showed a Tafel slope of 109 mV dec^−1^, significantly lower than that for CuAg/rGO (184 mV dec^−1^), suggesting faster ORR kinetics. Additionally, a higher diffusion current density was obtained for CoAg/rGO (−2.63 mA cm^−2^) than for CuAg/rGO (−1.74 mA cm^−2^). The average value of the number of electrons transferred during ORR was 2.8 for CuAg/rGO and 3.3 for CoAg/rGO electrocatalyst, further confirming the higher ORR activity of the latter. On the other hand, CuAg/rGO showed higher peak current densities (−3.96 mA cm^−2^) for HPRR compared to those recorded for CoAg/rGO electrocatalyst (−1.96 mA cm^−2^).

## 1. Introduction

As the energy demand increases over the years, the amount of available fossil fuels decreases. Furthermore, the carbon emissions and other negative environmental impacts of using fossil fuels deepen the need for clean energy sources. That is why electrochemical energy conversion and storage devices like fuel cells and batteries, which are green, renewable, and have high energy density output, are essential.

Both oxygen reduction reaction (ORR) and hydrogen peroxide reduction reaction (HPRR) are common cathodic half-reactions in fuel cells. ORR can proceed via either a two-electron or a four-electron pathway, depending on the type of electrocatalyst and electrolyte composition. The former is more sluggish, as it takes place in two steps—hydrogen peroxide formation and the hydrogen peroxide reduction reaction (HPRR), whereas the latter, the one-step process, is favored [1,2,3].

Regardless of the reaction pathway, energy investment is essential for diffusion, adsorption, desorption, bond breakage, and bond formation to occur and transform O_2_ into the reaction products (OH^−^ or H_2_O). Furthermore, the need to break the high-energy O=O bond increases the energy barrier, making this reaction at least ten times slower than HER, which is simultaneously occurring at the cathode. Consequently, it requires high overpotentials and cannot proceed without an electrocatalyst [4].

Since H_2_O_2_ is a reaction intermediate in ORR, it can (under certain conditions) be the final product of a two-electron reduction process [5,6,7]. Ideally, the catalysts for ORR should be active for the HPRR. Furthermore, although oxygen is an available and affordable reactant, HPRR has certain advantages over ORR. Namely, as a two-electron process with faster kinetics and higher electrode potential, it results in a higher voltage of the system. Utilizing liquid reactants instead of gaseous ones facilitates handling, storage, and transport and eliminates the need for complicated fuel cell construction and gas management [2]. Thus, HPRR-based fuel cells can have lower activation energy and better stability, ease of handling, and storage than those based on ORR [8]. HPRR can proceed by a direct mechanism, involving a two-electron reduction to water, which is preferred, or by an indirect mechanism that involves H_2_O_2_ decomposition to O_2_ and its subsequent reduction [1].

Generally, platinum group metal (PGM)-based catalysts were used for ORR due to their ability to decrease the activation energy and the required critical oxygen concentration. Still, high cost, an easy poisoning limit, and scarcity limit their applicability [9,10]. Consequently, many transition metals [11,12,13], metal oxides [14,15,16], metal-organic frameworks (MOFs) [17,18], zeolitic imidazolate frameworks (ZIFs) [17,18], and carbon-based [19,20,21] catalysts have emerged as efficient and robust low-cost alternatives to PGM. Concerning HPRR, the most potent catalysts can be divided into three categories—enzymes, noble metals (PGM and Ag), and macrocycle complexes of transition metals. However, the downsides of the first and the last are their low chemical, physical, or thermal stability, and the noble metals’ high price and the catalysis of H_2_O_2_ decomposition decrease their applicability [22]. The need for low-cost, stable, biocompatible materials with high catalytic activity for HPRR has resulted in the production of transition metal oxide-based materials [8,22,23,24] and carbon materials [25,26,27].

In this work, CuAg and CoAg-doped reduced graphene oxide (rGO) are proposed as efficient electrocatalysts for ORR and HPRR in alkaline media. Alkaline media, as a less corrosive environment compared to acidic media, allows using non-noble metal catalysts for ORR [28,29]. Additionally, ORR kinetics in alkaline media have been reported to be faster than in acidic media [30,31]. Thus, ORR can proceed at lower overpotentials in alkaline media (e.g., NaOH, KOH) than in several acidic media (e.g., H_2_SO_4_, H_3_PO_4_, HClO_4_). The superior performance of Pt electrocatalysts in alkaline media (KOH) than in acidic media (e.g., H_3_PO_4_) was attributed to the minimal adsorption of the OH^−^ ion in case of the KOH solution [31,32].

Apart from being more abundant and less expensive than PGM, silver exhibits good activity toward oxygen reduction [9]. Copper and cobalt are proven to enhance the physical and chemical properties of the catalyst upon addition, including the increase in the number of active sites, specific surface, and conductivity [10,17]. Andrade et al. [17] have shown that MOF-derived N-doped carbon with cobalt and copper (M-NC–CoCu) has fast ORR kinetics with an onset potential, E_onset_, of 0.85 V, a half-wave potential, E_1/2_, of 0.75 V, and a small Tafel slope (63 mV dec^−1^) in KOH. Under the same experimental conditions, the values for Pt/C were an E_onset_ of 0.93 V, an E_1/2_ of 0.82 V, and a Tafel slope of 70 mV dec^−1^.

It has been well-documented that bimetallic nanoparticle electrocatalysts containing a noble metal (e.g., Pt, Au, Ag) and a transition metal (e.g., Co, Cu) overcome the performance of the individual components for the ORR due to the improvement of the electronic properties [33,34]. For instance, Verma et al. [9] proved that the Ag-Cu bimetallic particle formation enhances the catalytic properties of the material, which can be explained by DFT calculations showing that Cu to Ag charge transfer results in the stronger material’s interaction with oxygen. Yin et al. [35] demonstrated that a catalyst containing both Ag and Cu particles exhibits a seven-fold enhancement of ORR activity compared to nanoporous Ag (NP-Ag). The former catalyst had an E_1/2_ of ca. 0.82 V and a Tafel slope of 100 mV dec^−1^, whereas that of NP-Ag and Pt/C was 145 mV dec^−1^ and 111 mV dec^−1^, respectively. The results were supported by presenting XRD and XPS data showing that the d-band center of Ag moves closer to the Fermi level owing to the alloying effect from Cu. These conclusions extrapolate to other transition metals, especially 3d elements with fairly good conductivity and oxygen absorption properties, including cobalt. Carbon-based materials not only exhibit good catalytic activity for both HPRR and ORR, but the implementation of rGO in the bimetallic material also increases the specific surface area, improves thermal stability and electrical and thermal conductivity of the material, and heightens carrier mobility and adsorptive properties [36,37]. Therefore, the proposed materials are expected to show excellent activity for ORR.

## 2. Materials and Methods 

Hydrazine hydrate (N_2_H_4_.xH_2_O, 78 wt.%), cobalt chloride (CoCl_2_·6H_2_O, 97 wt.%), copper chloride (CuCl_2_·2H_2_O, 97 wt.%), L-ascorbic acid (C_6_H_8_O_6_, 99 wt.%), sodium borohydride (NaBH_4_, 99 wt.%), silver nitrate (AgNO_3_, 99 wt.%), potassium permanganate (KMnO_4_, 99 wt.%), Nafion (5 wt.%), methanol (CH_3_OH, 97 wt.%), ethanol (C_2_H_5_OH, 99.8 wt.%) sodium hydroxide (NaOH, 97 wt.%), and potassium hydroxide (KOH, 85 wt.%) were obtained from Sigma-Aldrich (Burlington, MA, USA). The aqueous solutions were prepared by using distilled water.

Graphene oxide (GO) was synthesized by a modified Hummer’s method [38]. Namely, GO was prepared from graphite, where 2 g of graphite powder was dispersed in 100 mL concentrated H_2_SO_4_. This solution was cooled at 0 °C, and then 8 g of KMnO_4_ was gradually added with a constant stirring at 30 °C for 1 h. Next, 300 mL distilled water was added to the solution with a continuous stirring for 30 min at 90 °C. 400 mL distilled water and 6 mL H_2_O_2_ (30 wt.%) were added to the solution, where the solution color changed from dark brown to yellow-green. The obtained GO was separated by centrifuging and dried at 80 °C.

Reduced graphene oxide (rGO) was obtained in the second synthesis phase with N_2_H_4_.xH_2_O as a reducing agent. Namely, 100 mg GO and 100 mL distilled water were mixed. This brown-yellow solution was left in the ultrasound bath at 150 W until this solution stayed without visible particles. Then 1 mL of 32.1 M N_2_H_4_.xH_2_O was added, and the solution was heated at 100 °C, under a condenser, for further 24 h. The rGO was then separated by filtration as a black precipitate. After filtration, the rGO was washed 5 times with 100 mL distilled water and 5 times with 100 mL CH_3_OH, and then dried.

The procedure described by Krishna et al. [39] was followed with some changes. A total of 35 mg rGO was well dispersed in 11.6 mL distilled water, and then it was mixed with 167 mg CoCl_2_·6H_2_O (or 172 mg CuCl_2_·2H_2_O) by magnetic stirring for 30 min at room temperature. Then, 117 mg of L-ascorbic acid was added to the above solution and stirred for 10 min. Additionally, 5 mL of 1 M NaBH_4_ + 0.1 M NaOH solution was slowly added (dropwise) until the hydrogen was completely released. The obtained product was washed with distilled water and CH_3_OH, respectively.

This product was again redispersed in distilled water by sonication for 1 h. A total of 60 mg AgNO_3_ was mixed with obtained dispersion by mechanically stirring for 5 min. Then, 17 mg of L-ascorbic acid was added to the solution with 1 mL of 1 M NaBH_4_ + 0.1 M NaOH solution to reduce Ag ions. The CuAg/rGO and CoAg/rGO products were washed several times with distilled water and dried in the oven at 80 °C for 4 h.

Fourier transform infrared spectroscopy (FTIR, Perkin Elmer Spectrum One Spectrometer, Waltham, MA, USA) and Raman spectroscopy (Leica Microsystems GmbH, Wetzlar, Germany) were used to determine surface functional groups of the rGO, CuAg/rGO, and CoAg/rGO electrocatalysts. X-ray diffraction analysis (XRD) using a Rigaku Ultima IV diffractometer (Rigaku, Japan) in Bragg-Brentano geometry, with Ni-filtered CuKα radiation (λ = 1.54178 Å) was used for examining the structure of CuAg/rGO and CoAg/rGO electrocatalysts. The morphology and microstructure of CuAg/rGO and CoAg/rGO electrocatalysts were examined by transmission electron microscopy (TEM) using a HITACHI H-8100 microscope (Hitachi, Tokyo, Japan). Scanning electron microscopy with integrated energy-dispersive X-ray spectroscopy (SEM-EDS) detector was done with a scanning electron microscope Phenom™ ProX Desktop SEM (ThermoFisher Scientific™, Waltham, MA, USA) to examine the surface morphology and atomic composition of CoAg/rGO and CuAg/rGO electrocatalysts. Cu, Co, and Ag wt.% in the two samples were determined by inductively coupled plasma with optical emission spectroscopy (ICP-OES) using an ICP optical emission spectrometer Optima 7000DV (Perkin Elmer, Waltham, MA, USA).

A total of 5 mg of powdered CuAg/rGO or CoAg/rGO were mixed with 980 µL of C_2_H_5_OH and 20 µL of Nafion (5%) and left in an ultrasonic bath for 1 h. After that, 14 µL of the prepared catalytic ink was pipetted onto a glassy carbon rotating disk electrode (RDE, 0.19625 cm^2^). The electrode was left to dry at room temperature.

Ivium VO1107 potentiostat/galvanostat (Ivium Technologies B.V., Eindhoven, The Netherlands) was used for all electrochemical studies in a three-electrode system. A saturated calomel electrode (SCE, HI5412, Hanna Instruments) and a graphite rod (Sigma-Aldrich, 99.995 wt.%) were employed as reference and counter electrodes, respectively. The supporting electrolyte was 0.1 M KOH. As the SCE can suffer from instability in highly alkaline media, the potential difference between the herein used SCE and unused SCE was regularly measured using a multimeter, to ensure the quality of SCE potential measurements. All potentials in this work were converted to the reversible hydrogen electrode (RHE) scale using the following equation: E_RHE_ = E_SCE_ + 0.242 V + 0.059 V × pH. Current densities were calculated using the geometric area.

Cyclic voltammograms (CVs) of CuAg/rGO and CoAg/rGO electrodes were recorded from 0.86 to 0.96 V at different polarization rates ranging from 5 to 100 mV s^−1^ in 0.1 M KOH solution saturated with nitrogen (N_2_, 99.995 vol.%, Messer).

For ORR studies, linear sweep voltammograms (LSVs) were recorded in a potential range from 0.2 to 1 V at a scan rate of 20 mV s^−1^ in 0.1 M KOH solution saturated with oxygen (O_2_, 99.995 vol.%, Messer) at room temperature. ORR rotating-disk electrode (RDE) measurements were carried out at different rotation rates from 400 to 3600 rpm. Current densities obtained in N_2_-saturated solutions were subtracted from those measured in O_2_—saturated solutions. Stability tests of CuAg/rGO and CoAg/rGO electrocatalysts were done in 0.1 M KOH O_2_—saturated solutions at 0.6 V for 4 h. HPRR LSVs were recorded in a potential range from 0.2 to 1 V at a scan rate of 20 mV s^−1^ in 0.05 M H_2_O_2_ + 0.1 M KOH solution saturated with N_2_.

## 3. Results and Discussion

### 3.1. Characterization of CuAg/rGO and CoAg/rGO Electrocatalysts

**Figure 1:**
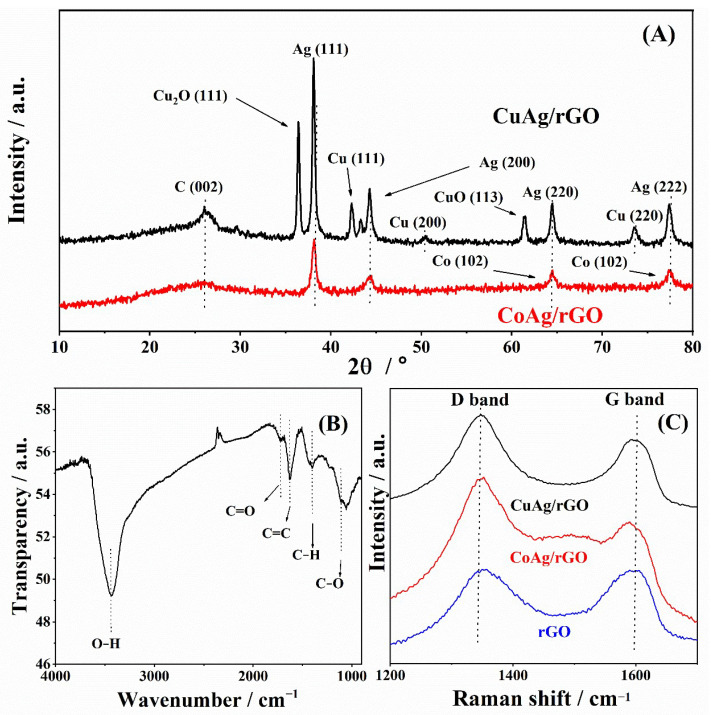
XRD patterns of CuAg/rGO and CoAg/rGO electrocatalysts (**A**). FTIR of rGO (**B**) and Raman spectra of rGO, CuAg/rGO, and CoAg/rGO electrocatalysts (**C**).

The XRD analysis of CuAg/rGO and CoAg/rGO electrocatalysts is presented in Figure 1A. The peak at about 26° was observed at both XRD patterns of CuAg/rGO and CoAg/rGO electrodes as a reflection of the C (002) plane [40]. Four characteristic diffraction peaks of bulk metallic Ag at 2θ of ca. 38.3°, 44.4°, 64.5°, and 77.5° were noticed for both electrodes as the diffraction from crystal planes of Ag (111), (200), (220), and (222), respectively [40,41,42]. The XRD pattern of the CuAg/rGO electrode showed a diffraction peak of the Cu_2_O(111) plane at ca. 36° and three diffraction peaks of Cu (111), (200), and (220) crystal planes at 2θ of ca. 42.3°, 50.4°, and 73.6°, respectively [43]. Two diffraction peaks of Co appeared at 2θ of ca. 64.5 and 77.5°, corresponding to the reflections of the Co (102) and (103) planes, respectively [44].

The FTIR spectrum of rGO is presented in Figure 1B, where a broad peak was noticed at 3438 cm^−1^, corresponding to the OH^−^ groups of water adsorbed on rGO [45,46]. The adsorption peaks at 1724, 1623, and 1103 cm^−1^ were visible for the characteristic carboxyl group C=O, an aromatic C=C group, and a C–O group, respectively [45,46].

The Raman spectra of rGO, CuAg/rGO, and CoAg/rGO (Figure 1C) show two characteristic strong peaks, known as D and G bands, at 1351 and 1595 cm^−1^ for rGO, at 1346 and 1591 cm^−1^ for CuAg/rGO, and at 1350 and 1596 cm^−1^ for CoAg/rGO [45]. The G band corresponds to the bond extending of all pairs of sp^2^ atoms, whereas the D band represents the presence of structural defects, crystal imperfections, and the measure of the degree of disorder of carbon-based materials [45,46]. The 2D called G’ band characteristic for graphite materials was noticed at 2704 cm^−1^ [45,47,48]. The D + G peak also related to disorder with structure was obtained at 2933 cm^−1^ [45,47,48]. The intensity ratio of the D and G bands (I_D_/I_G_) was found to be 0.85 for rGO, CuAg/rGO, and CoAg/rGO. The broad, low-intensity peak at about 1483 cm^−1^ for CoAg/rGO could be related to the specific structure of Ag_2_O [49]. These results utterly confirmed the presence of an rGO structure in CuAg/rGO and CoAg/rGO electrocatalysts.

**Figure 2:**
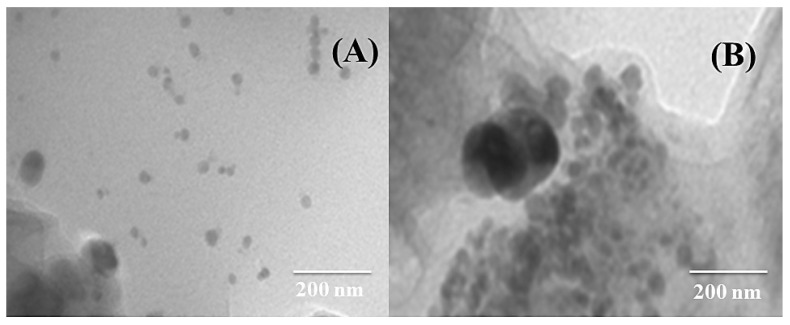
TEM images of CuAg/rGO (**A**) and CoAg/rGO (**B**) electrocatalysts.

Figure 2A,B shows TEM images of CuAg/rGO and CoAg/rGO electrocatalysts, revealing the formation of metal nanoparticles.

**Figure 3:**
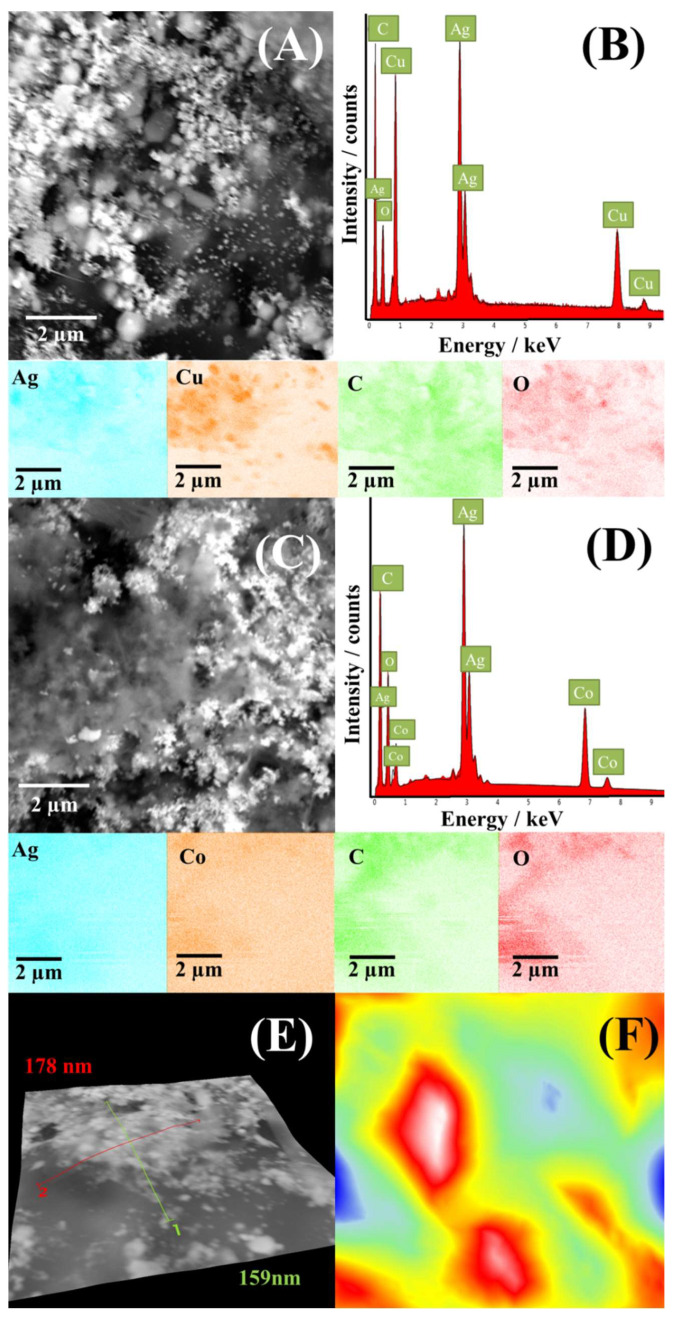
SEM images of CuAg/rGO (**A**) and CoAg/rGO (**C**) electrocatalysts with the corresponding EDS spectrum of CuAg/rGO (**B**) and CoAg/rGO (**D**) and mapping images of Ag, Cu, Co, C, and O with the corresponding 3D SEM surface reconstruction (**E**,**F**).

SEM images of CuAg/rGO and CoAg/rGO electrocatalysts with corresponding EDS spectra and mapping are presented in Figure 3. SEM images of CuAg/rGO and CoAg/rGO (Figure 3A,C) show a uniform distribution of metal nanoparticles (bright parts) over rGO (gray parts). Corresponding EDS spectra and mapping (Figure 3B,D) show the presence of Ag, Cu, Co, C, and O elements and confirm their uniform distribution.

The elemental composition of the electrocatalysts determined by ICP-OES shows 27.8 and 31.5 wt.% of Ag and Cu, respectively, for CuAg/rGO and 27.0 and 17.3 wt.% of Ag and Co, respectively, for CoAg/rGO. Though the present study was “conceived” as an initial study to confirm or disproof AgCo and/or AgCu on rGO as electrocatalysts for the ORR and to point out the path that further research should take, it should be noted that the electrocatalytic performance of bimetallic nanoparticles is governed by their composition, among other factors, including particle size and surface oxidation state [33,50,51].

**Figure 4:**
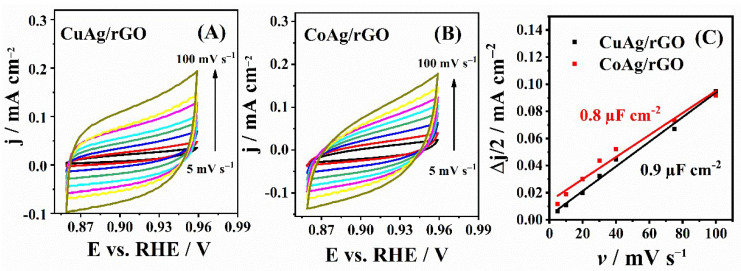
CVs of CuAg/rGO (**A**) and CoAg/rGO (**B**) electrocatalysts and the corresponding double-layer capacitance plots (**C**) in N2-saturated 0.1 M KOH solution at different scan rates.

Electrochemical double-layer capacitance, C_dl_, was calculated from cyclic voltammograms (CVs) of CuAg/rGO and CoAg/rGO electrocatalysts (Figure 4) recorded in N_2_-saturated 0.1 M KOH solution at different scan rates. C_dl_ was found to be 0.9 and 0.8 µF cm^−2^ for CuAg/rGO and CoAg/rGO, respectively, with these values being directly proportional to the electrochemically active surface area (ECSA) [52,53]. The somewhat higher ECSA of CuAg/rGO than CoAg/rGO electrocatalyst represents the higher number of active sites for ORR and HPRR [52,53]. It should be mentioned that high-surface-area rGO contributes to the non-faradaic, capacitive currents and, consequently, affects the accurate ECSA determination. Still, the present ECSA estimation by the C_dl_ method is suitable for the ECSAs comparison, as both materials contain rGO. Moreover, the C_dl_ method for estimating alloys’ ECSA considers all the alloy components [54,55].

Regarding the nature of active sites in the two electrocatalysts, the Ag(111) plane has been pointed out as highly active toward ORR, where the presence of different defects further increases the surface’s activity for ORR [56]. The higher availability of active sites for ORR on Ag(111) is attributed to the weaker OH^−^ adsorption. Furthermore, Co (moiety) has been presented as an important active site for ORR in alkaline media [57,58,59,60]. Cu shows high theoretical ORR activity in the volcano plot and has four valence states (Cu(I), Cu(II), Cu(0), and Cu(IV)) which contribute to ORR electrocatalysis [61]. Finally, the presence of rGO also brings different types of surface active sites for ORR, including unsaturated carbons defect sites and carbon-oxygen polar groups.

### 3.2. ORR Activity of CuAg/rGO and CoAg/rGO Electrocatalysts

**Figure 5:**
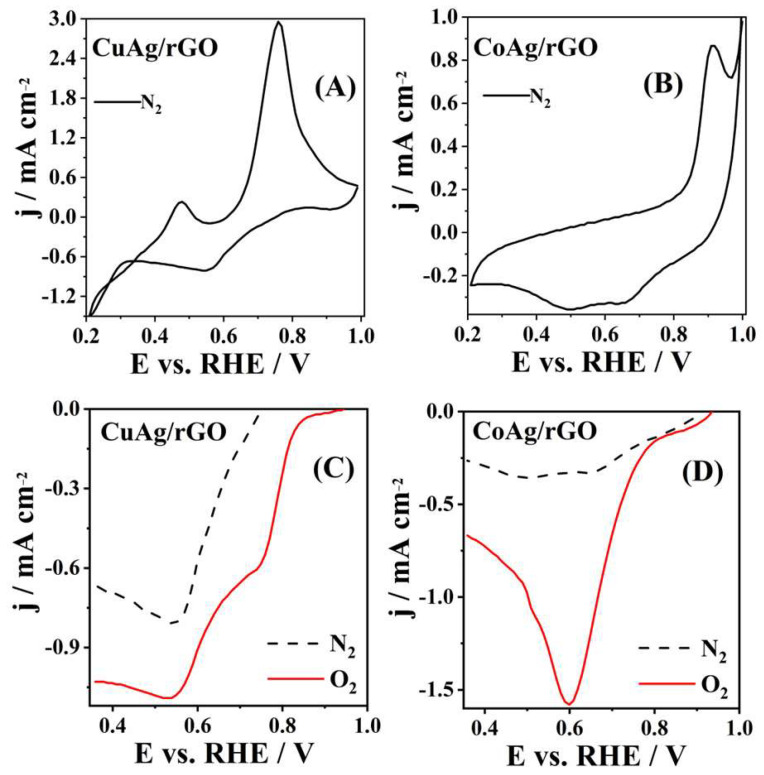
CVs of CuAg/rGO (**A**) and CoAg/rGO (**B**) electrocatalysts recorded at 20 mV s^−1^ in N_2_-saturated 0.1 M KOH solution and the cathodic part of CVs of CuAg/rGO (**C**) and CoAg/rGO (**D**) electrocatalysts recorded at 20 mV s^−1^ in N_2_- and O_2_-saturated 0.1 M KOH solution.

CVs of CuAg/rGO and CoAg/rGO electrocatalysts recorded in N2-saturated solution are presented in Figure 5A,B. Two well-defined anodic peaks appearing at 0.47 and 0.76 V (Figure 5A) are attributed to the oxidation of the CuAg/rGO surface, where the first anodic peak presents the oxidation of Cu(0) to Cu(I), and the second anodic peak presents the oxidation of Cu(I) to Cu(II) [62,63]. An additional cathodic peak was noticed at 0.55 V corresponding to the reduction of Cu(II) to Cu(I) [62,63]. The CoAg/rGO electrocatalyst showed one anodic peak at 0.92 V (Figure 5B) corresponding to the oxidation Co(II) to Co(III) [64,65]. Figure 5C,D presents ORR polarization curves of CuAg/rGO and CoAg/rGO electrocatalysts, where both materials show ORR activity in 0.1 M KOH. The CoAg/rGO electrocatalyst exhibited a current density of −1.56 and −0.34 mA cm^−2^, at 0.6 V, in O_2_ and N2-saturated solution, respectively (Figure 5D). The current density of −0.58 and −0.03 mA cm^−2^, at 0.7 V, for the first cathodic peak and −1.1 and −0.82 mA cm^−2^, at 0.5 V, for the second cathodic peak were noticed for the CuAg/rGO electrocatalyst in O_2_ and N2-saturated solution, respectively (Figure 5C). CuAg/rGO and CoAg/rGO gave about 20 (first reduction peak) and 5 times higher cathodic current densities, respectively, in O_2_−saturated than in deaerated solution (Figure 5C,D). Even 50% times higher ORR current was observed for CoAg/rGO than for the CuAg/rGO electrocatalyst. An Ag/Co/C hybrid electrocatalyst was recently pointed out as a promising candidate for ORR in alkaline media because of its excellent ORR activity and stability [13]. This behavior of Ag/Co/C during ORR was described by density functional theory (DFT), where ORR starts due to the strong interaction between Co and O in the first step, and the following steps on the Ag surface occur with a small barrier [13]. Additionally, Verma et al. showed that oxygen adsorption on Ag clusters increased by doping with copper atoms, confirmed by binding energy DFT calculations [9].

**Figure 6:**
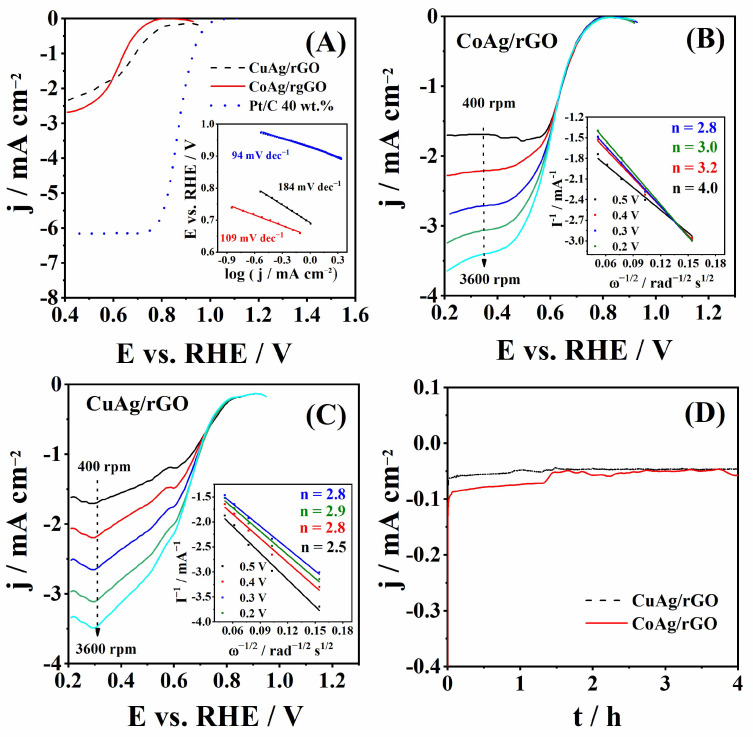
ORR polarization curves measured at 20 mV s^−1^ and 1600 rpm with the corresponding Tafel plots shown in the inset (**A**), ORR polarization curves of CoAg/rGO (**B**) and CuAg/rGO (**C**) electrocatalysts at different rotation rates with Koutecky-Levich analysis in the inset, and the chronoamperometric curves (**D**) recorded in O_2_-saturated 0.1 M KOH solution.

ORR polarization curves of CuAg/rGO and CoAg/rGO electrocatalysts obtained at 1600 rpm in O_2_-saturated solution are presented in Figure 6A. E_onset_ and E_1/2_ were found to be 0.81 and 0.71 V for CuAg/rGO and 0.76 and 0.62 V for CoAg/rGO, respectively. Thus, CuAg/rGO showed 50 and 90 mV more positive ORR E_onset_ and E_1/2_, respectively, compared to the CoAg/rGO electrocatalyst. Ag@CuO nanoparticles showed E_1/2_ of 0.74 V [66], which is ca. 30 mV more positive than the herein examined CuAg/rGO electrocatalyst.

The ORR kinetics was examined by Tafel analysis of the curves presented in Figure 6A. Tafel slopes of 184 and 109 mV dec^−1^ for CuAg/rGO and CoAg/rGO were calculated. The CoAg/rGO electrocatalyst showed a lower Tafel slope than CuAg/rGO, indicating faster ORR kinetics. The big difference in Tafel slopes can be a consequence of differences in the physicochemical properties of the CuAg/rGO and CoAg/rGO surface, such as oxygen coverage and the number of active sites [56]. Nanoporous Ag (NP-Ag) [35] and monometallic Ag nanoparticles (Ag NPs) [67] showed a Tafel slope of 145 and 113 mV dec^−1^ during ORR in 0.1 M KOH, respectively. A Tafel slope of 96 mV dec^−1^ was determined for Ag/OCPN at the low current density region [68]. S. Zoladec et al., determined Tafel slopes of 100 and 120 mV dec^−1^ for silver nanoparticles deposited on rGO-carboxylate and rGO-SiO_2_, respectively [56]. Five different nanoporous AgCu (NP-AgCu) catalysts gave Tafel slopes ranging from 100 to 124 mV dec^−1^ in 0.1 M KOH [35]. CuO nanoparticles (CuO) and CuO nanoparticles on graphene (CuO/G) showed a Tafel slope of 207 and 141 mV dec^−1^, respectively [69].

Furthermore, a higher diffusion current density, j_d_, was obtained for CoAg/rGO (−2.63 mA cm^−2^) than for the CuAg/rGO (−1.74 mA cm^−2^) electrocatalyst. Ag/C gave a j_d_ of ca. −1.8 mA cm^−2^ than herein tested silver electrocatalysts in alkaline media [70]. A j_d_ of −2.8 mA cm^−2^ was obtained for Ag/Co_3_O_4_–C composite in 1 M KOH, comparable with the j_d_ obtained for CoAg/rGO [70]. ORR performance of two prepared electrocatalysts was further compared to that of commercial Pt/C (40 wt.% Pt), Figure 6A. As expected, higher current densities were recorded with the Pt/C electrode, along with a Tafel slope of 94 mV dec^−1^. However, the significantly lower price of the herein prepared electrocatalysts and the possibility of further improvement of their performance justify their use for ORR studies.

Figure 6B,C shows the ORR polarization curves of CoAg/rGO and CuAg/rGO electrocatalysts, respectively, at different rotation rates, ranging from 400 to 3600 rpm. From these data, Koutecky-Levich analysis calculated the number of exchanged electrons, *n*, during ORR in alkaline media [41]. The value of *n* was found to range from 2.8 to 4.0 and from 2.5 to 2.9 for CoAg/rGO and CuAg/rGO electrocatalysts (inset of Figure 6B,C), respectively, suggesting that ORR occurred by the mixed 2e^−^ and 4e^−^ reduction pathway. [70,71]. It is worth noting that at a more positive potential, four electrons were transferred during ORR at CoAg/rGO. The transfer of four electrons during ORR at Ag-based electrocatalysts often involves a 2e^−^ + 2e^−^ series mechanism rather than a direct 4e^−^ mechanism. Thus, the HPRR activity of the prepared electrocatalysts was also probed.

**Figure 7:**
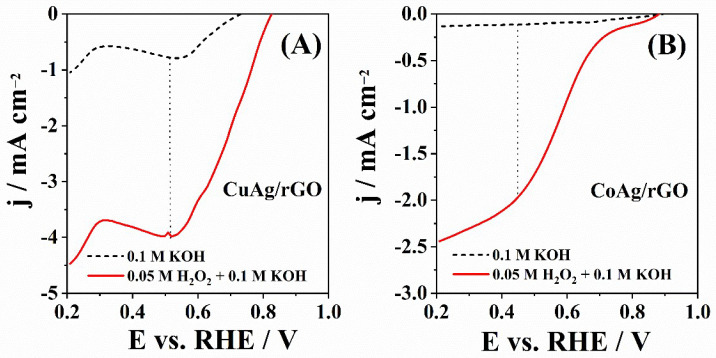
LSVs of CuAg/rGO (**A**) and CoAg/rGO (**B**) electrocatalysts recorded at 20 mV s^−1^ in N_2_-saturated 0.1 M KOH solution without (--) and with (-) 0.05 M H_2_O_2_.

Figure 7 shows LSVs of CuAg/rGO and CoAg/rGO electrocatalysts recorded in 0.1 M KOH solution as a supporting electrolyte in the absence and presence of H_2_O_2_. Both materials showed electrocatalytic activity for HPRR with a reduction peak at ca. 0.50 and 0.45 V for CuAg/rGO and CoAg/rGO electrocatalysts, respectively. Carbon paste electrodes modified by Ag-supported carbon microspheres (Ag-CMS/CPE) obtained by the hydrothermal method showed a similar HPRR peak potential value in alkaline media [72]. CuAg/rGO showed a higher peak current density of −3.96 mA cm^−2^ than CoAg/rGO electrocatalyst (−1.96 mA cm^−2^).

It should be mentioned that the ORR at the currently examined CoAg/rGO electrocatalyst led to a higher *n* than at Ag/C, with 2.7 electrons exchanged during ORR in alkaline media [70]. Ag@C nano cables delivered 3.3 electrons during ORR in 0.1 M KOH [71]. Silver and copper nanocatalysts supported with multi-walled carbon nanotube (AgCu/MWCNT) delivered 3.5 electrons during ORR in alkaline media [73]. Table 1 compares the ORR parameters determined for CuAg/rGO and CoAg/rGO electrocatalysts with that previously reported for similar materials.

To confirm the stability of the electrocatalysts’ activity towards ORR in alkaline media, chronoamperometric curves of CuAg/rGO and CoAg/rGO electrocatalysts were run for 4 h (Figure 6D). Both electrocatalysts showed good stability for ORR. Current densities of −0.046 and −0.055 mA cm^−2^ were obtained for CuAg/rGO and CoAg/rGO at 4 h, representing ca. 28 and 29% decrease of the corresponding initial values. For both electrocatalysts, the decrease was observed within the first 200 s, with fairly constant current densities for the rest of the measurement. Thus, the authors believe this decrease is caused by the adsorption of impurities on the surface of the electrocatalysts rather than by the metal nanoparticles’ agglomeration or detachment [34].

## 4. Conclusions

Silver copper and silver cobalt nanoparticles were deposited on synthesized rGO (CuAg/rGO and CoAg/rGO) and tested for ORR in alkaline media. Raman spectroscopy clearly showed that rGO was obtained during synthesis, and the surface morphology and atomic composition of CuAg/rGO and CoAg/rGO electrocatalysts were confirmed by SEM-EDS analysis. The average particle diameter obtained by TEM analysis for CuAg/rGO and CoAg/rGO electrocatalysts was 5.7 and 5.5 nm, respectively. CoAg/rGO showed a lower Tafel slope than the CuAg/rGO electrocatalyst. The lower Tafel slope, higher ORR current densities, j_d_, and *n* of CoAg/rGO demonstrate faster ORR kinetics and better ORR catalytic activity than the CuAg/rGO electrocatalyst. Additionally, both electrocatalysts showed reasonable activity for HPRR, with a reduction peak at ca. 0.50 and 0.45 V for CuAg/rGO and CoAg/rGO, respectively. These electrocatalysts can potentially replace high-cost platinum group metal-based catalysts because of their easy, fast, and low-cost synthesis, coupled with their good ORR activity and stability in alkaline media.

## Figures and Tables

**Table 1:** Comparison of ORR parameters of CuAg/rGO and CoAg/rGO electrocatalysts with the activity of similar materials reported in the literature.

ORR Catalysts	j_d_@(1600 rpm) /mA cm^−2^	E_onset_/V	E_1/2_/V	*n*	Source
CuAg/rGO	−1.74	0.81	0.71	2.5–2.9	This work
CoAg/rGO	−2.63	0.76	0.62	2.8–4.0	This work
Ag_9_Cu_1_/MWCNT *	−2.7	−	−	3.5	[73]
Ag/C	−1.8	−	−	2.7	[70]
Co/C	−2.48	0.88	0.83	−	[13]
Ag@CuO	−	−	0.74	3.8	[66]
Ag@C nanocables	−2.5	0.75	−	3.3	[71]
Ag/Co_3_O_4_–C	−2.8	0.84	0.78	3.8	[70]
Ag/CPN **	−4.8	0.83	−	3.7	[68]
Ag/OCPN ***	−5.4	0.87	−	4.0	[68]

* MWCNT—multi-walled carbon nanotubes, ** CPN—carbonaceous polypyrrole nanotubes, *** OCPN—oxygen-doped carbonaceous polypyrrole nanotubes.

## Data Availability

Data available on request to the corresponding author.
